# Determinants of Unsupervised Medical Termination of Pregnancy Pill Usage Among Women: A Cross-Sectional Study From North India

**DOI:** 10.7759/cureus.49321

**Published:** 2023-11-24

**Authors:** Reena Pal, Puneet K Gupta, Stuti Tyagi, Himani Palariya, Vidhi Vora, Pratik Agarwal

**Affiliations:** 1 Obstetrics and Gynaecology, Government Doon Medical College, Dehradun, IND; 2 ICFAI Business School, The Institute of Chartered Financial Analysts of India (ICFAI) University, Dehradun, IND; 3 Medicine, Lokmanya Tilak Municipal Medical College and Sion Hospital, Mumbai, IND; 4 Research, PearResearch, Dehradun, IND; 5 Medicine, Lokmanya Tilak Municipal Medical College, Mumbai, IND

**Keywords:** maternal mortality, female reproductive health, unsafe abortion, unsupervised mtp pill, gynecology, abortion india, abortion pill

## Abstract

Background

Medical termination of pregnancy (MTP) pills, primarily comprising mifepristone and misoprostol, have proven highly effective and safe under medical supervision. However, unsupervised MTP pill use is on the rise, posing serious health risks. Unsafe abortions remain a global public health concern, with a high incidence in developing countries like India.

Methods

We conducted a cross-sectional study at a tertiary healthcare center in India from February to April 2023. We enrolled 150 women with a history of unsupervised MTP pill use. Data were collected using structured questionnaires, including demographic information, awareness, sources of information, reasons for self-medication, and knowledge of complications.

Results

The majority of participants (50%) were aged 25-29 years. Low-income women (<3000pc) constituted 46.66% of the sample. Husbands played a significant role in advocating MTP pill use (57.33%). Ninety percent of pills were obtained directly from pharmacies. Shockingly, 97.3% of women were unaware of MTP pill complications, and 84% did not follow the recommended regimen. Significant associations were found between income, religion, education, age, parity, and reasons for self-medication, as well as recommendations for MTP pill use.

Conclusion

Our study revealed a diverse demographic of women seeking unsupervised MTP pill intake. Low-income women were disproportionately affected, emphasizing the need for improved healthcare access and education. Husbands played a crucial role in advocating MTP pill use, highlighting the importance of including men in reproductive health discussions. Lack of awareness and non-adherence to recommended regimens posed substantial risks. To combat unsafe abortions, a multifaceted approach is needed. Reproductive health education, regulatory measures, improved healthcare accessibility, and tailored interventions are essential.

## Introduction

Medical termination of pregnancy (MTP) pills, typically comprising a combination of drugs, most commonly mifepristone followed by misoprostol, have demonstrated a high success rate (93%-98%) in safely and effectively terminating pregnancies when administered under medical supervision [1]. According to WHO guidelines, women seeking a medical abortion should confirm their pregnancy, accurately determine their gestational age, identify the location of the pregnancy, and rule out any contraindications. It is also recommended that the procedure be performed by a qualified healthcare provider or facility with access to backup resources in the event that the procedure is unsuccessful or incomplete [2]. However, a growing trend of unsupervised MTP pill use has emerged, where women choose to take these medications without direct guidance or medical oversight from qualified healthcare providers. While unsupervised intake offers women more autonomy and privacy, it also raises serious concerns about the procedure's safety and effectiveness. In such cases, the lack of medical supervision may result in life-threatening complications such as severe bleeding, infection, and sepsis caused by incomplete abortions, failed abortions, and ruptured ectopic pregnancies [3,4].

Unsafe abortions, resulting from unwanted or unplanned pregnancies, are a persistent and serious global public health concern, contributing significantly to high maternal mortality and morbidity rates. According to the World Health Organization (WHO), every eight minutes, a woman in a developing country loses her life due to complications arising from an unsafe abortion [1]. The term "unsafe abortion" refers to procedures for terminating unintended pregnancies performed by individuals who lack the necessary medical skills, or in settings that do not meet minimum medical standards, or both. Surprisingly, 97% of all unsafe abortions occur in developing countries, emphasizing the importance of addressing this issue in countries like India, as well as on a global scale [2].

This crisis is especially acute in the context of India. Annually, approximately 6.4 million abortions are performed in the country, with 56% of them classified as unsafe, accounting for 8%-20% of all maternal fatalities [3]. As concerning as these figures are, they only scratch the surface of the issue. The true number of induced abortions is likely much higher due to a significant percentage of unreported cases, exacerbated by years of unregulated sales of over-the-counter MTP pills [4]. Misuse of MTP pills by untrained individuals, traditional birth attendants, and unscrupulous providers, combined with widespread ignorance and a lack of awareness about potential complications, as well as easy over-the-counter access despite legal bans, elevate MTP pills to a significant public health concern, particularly in developing countries such as India, where healthcare access remains limited [5].

While there is extensive data on unsafe abortions, MTP pill use, complications, and management, there remains a significant gap in our understanding of the specific circumstances leading to unsupervised consumption. India's diverse population, complex legal and socio-cultural landscape, economic disparities, and unequal access to healthcare services all combine to create a unique and complex context in which to examine this pressing issue. We anticipate that conducting this research, particularly in a city with a high rate of unsafe abortions in India, will bridge this knowledge gap and shed light on the underlying factors fueling the unsupervised consumption of MTP pills. Our findings have the potential to inform strategies aimed at reducing the rate of unsafe abortions and promoting equitable and affordable healthcare policies for women in India and other developing countries facing similar challenges.

## Materials and methods

Study setting

This cross-sectional observational questionnaire-based study was conducted at Government Doon Medical College, situated in Dehradun, India, from February to April 2023. The study was carried out at the Department of Obstetrics and Gynecology, which serves as a vital healthcare facility catering to the diverse population in the region.

Ethical considerations

This study received ethical approval from the Institutional Review Board of Government Doon Medical College. Informed consent was obtained from all participants, ensuring that they were aware of the study's purpose and their voluntary participation. Confidentiality and anonymity of participants' responses were maintained throughout the study.

Participant selection

The study focused on women visiting the gynecological outpatient department during the specified period who had a history of self-administering medical termination pills (MTP). A total of 150 women were enrolled in the study after obtaining their written informed consent. Participants included in the study comprised women with a documented history of unsupervised, self-administration of MTP pills. Participants were excluded from the study if they had taken MTP pills under the direct supervision of a registered medical practitioner.

Data collection and analysis

Data for this study were collected using structured face-to-face questionnaires administered by trained research personnel. The recorded variables included demographic information, such as age, literacy status, occupation, socioeconomic status, marital status, and parity. Participants were also questioned about their awareness and knowledge of MTP pills, including their mode of action, indications, contraindications, and potential risks. Information on participants' previous experiences with unsupervised MTP pill intake and the source of pill procurement (e.g., registered medical practitioner, pharmacy) was documented. Additionally, a detailed history was collected to understand the reasons motivating participants to choose MTP pills for pregnancy termination, including both personal and situational factors. The data were analyzed using IBM SPSS Statistics for Windows, Version 26 (Released 2019; IBM Corp., Armonk, New York). Descriptive statistical analysis was performed, followed by appropriate statistical measures to calculate the inter-variable association.

## Results

Table 1 highlights the demographic and obstetric profile of the sample. The sample size (N=150) consisted of women above the age of 18 years, with 50% (n=75) of the population comprising individuals aged 25-29. The majority of the sample was educated, with 36% (n=54) being graduates and 28% (n=42) having passed secondary school. However, the majority (46.66%, n=70) had a per capita average family income of less than 3000 (Indian Rupees). In terms of parity, the population predominantly consisted of Para 1 or 2 women (62.66%, n=94), followed by Para 3 or more (20%, n=30), and nulliparous women (17.33%, n=26).

**Table 1:** Demographic and obstetric profile

Age (years)	Frequency (N=150)	Percentage (%)
18-24	10	6.66
25-29	75	50.00
30-34	46	30.66
>34	19	12.66
Educational level		
No formal education	20	13.33
Primary school	34	22.66
Secondary school	42	28.00
Graduation	54	36.00
Average family income per capita (in Indian Rupees)		
<3000pc	70	46.66
3000-5999pc	55	36.66
>6000pc	25	16.66
Religion		
Hindu	124	82.66
Muslim	26	17.33
Parity		
Nulliparity	26	17.33
Para 1-2	94	62.66
Para ≥3	30	20.00

Table 2 shows that 48.66% (n=73) of the sample had previous experience using the MTP pill. A majority (63.33%, n=95) received their information on MTP pill use from their husbands, followed by parents and friends (17.33%, n=26). Time-saving was stated as the primary reason for self-medication by many (58.90%, n=77), followed by easy availability (28.66%, n=43), cheaper cost of self-medication (8.00%, n=12), and long waiting times for healthcare services (7.33%, n=11). The direct source of medication was a pharmacy for 90% (n=135) of the study participants. In a majority of cases (57.33%, n=86), the recommendation for MTP pill consumption was made by the husband. A significant share of recommendations for pill consumption also came from the women themselves (18.66%, n=28) and pharmacists (16.00%, n=24). Surprisingly, the pill was not taken according to the regimen in 84% (n=126) of cases. A shocking 97.3% (n=146) of women were unaware of the complications of using the MTP pill.

**Table 2:** Knowledge and previous and current experience on self-medication

Previous use of MTP pill	Frequency (N=150)	Percentage (%)
Yes	73	48.66
No	77	51.33
Sources of information		
Radio/newspaper/TV	09	6.00
Health professional	10	6.66
Parents and friends	26	17.33
Husband	95	63.33
No information	10	6.66
Reason for self-medication		
Time saving	77	51.33
Easy availability	43	28.66
Self-medication is cheaper	12	8.00
Poor health services accessibility	07	4.66
Long waiting time for health services	11	7.33
Source		
Pharmacy	135	90.00
Health facility	15	10.00
Recommendation for MTP pill		
Herself	28	18.66
Husband/relatives	86	57.33
Pharmacist	24	16.00
Healthcare worker	12	8.00
MTP pill taken as recommended regime		
Yes	24	16.00
No	126	84.00
Awareness of complications		
Yes	4	2.70
No	146	97.3

**Figure 1:**
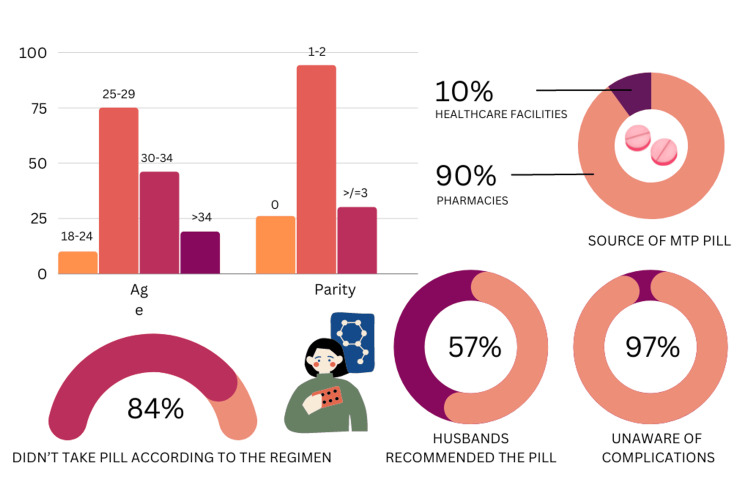
Understanding the determinants of unsupervised medical termination of pregnancy (MTP) pill usage among women

An income of less than 3000 per capita showed a statistically significant association with reasons for self-medication (p=0.000). Additionally, their age, educational level, 3000-6000 per capita average family income, and religion were significantly associated (p<0.05) with MTP pill self-medication. Tables 3, 4 show the association of demographic variables with reasons for self-medication and the recommendation of MTP pill use. Figure 1 provides a comprehensive synopsis of the determinants of unsupervised MTP pill use.

**Table 3:** Association with reasons for self-medication This table presents the association between the reason for self-medication and sociodemographic variables. A significant variation in the reason for self-medication was found to be related to average family income and religion.

	Reason for self-medication				
Variables	Time-saving/long waiting time for health services	Easy availability	Self-medication is cheaper	Poor health services accessibility	Chi-square, p-value
Age (years)					
18-24	5	2	1	2	13.81, 0.129
25-29	39	28	6	2	
30-34	32	10	3	1	
>34	12	3	2	2	
Educational level					
No formal education	14	3	2	1	4.027, 0.909
Primary school	19	11	2	2	
Secondary school	24	14	2	2	
Graduation	31	15	6	2	
Average family income per capita (in Indian Rupees)					
3000pc	52	15	2	1	41.38, 0.000
3000-5999pc	29	22	2	2	
>6000pc	7	6	8	4	
Religion					
Hindu	76	37	8	3	11.06, 0.011
Muslim	12	6	4	4	
Parity					
Nulliparity	17	4	3	2	10.97, 0.0892
Para 1-2	52	34	4	4	
Para ≥3	19	5	5	1	

**Table 4:** Association with recommendation for medical termination of pregnancy (MTP) pill This table depicts the association between recommendations for MTP pills and sociodemographic variables. The variation in recommendation is found to be significant with age, education level, average family income, parity, and religion.

	Recommendation for MTP pill				
Variables	Herself	Husband/relatives	Pharmacists	Healthcare worker	Chi-square, p value
Age (years)					
18-24	6	2	1	1	50.33, 0.000
25-29	15	52	4	4	
30-34	5	29	8	4	
>34	2	3	11	3	
Educational level					
No formal education	12	4	2	2	35.82, 0.000
Primary school	8	19	4	3	
Secondary school	6	27	5	4	
Graduation	2	36	13	3	
Average family income per capita (in Indian Rupees)					
3000pc	22	37	9	2	41.38, 0.000
3000-5999pc	5	39	8	3	
>6000pc	1	10	7	7	
Religion					
Hindu	24	79	14	7	20.13, 0.000
Muslim	4	7	10	5	
Parity					
Nulliparity	4	18	2	2	13.34, 0.039
Para 1-2	14	58	17	5	
Para ≥3	10	10	5	5	

## Discussion

The demographic characteristics of the study participants revealed important information about the population seeking unsupervised MTP pill intake. Notably, a wide range of women were represented, reflecting the issue's multifaceted nature. Understanding these differences is critical for tailoring interventions and policies to the specific needs of different demographic groups. Women aged 25-29 constituted the majority (50%) of the study's sample (N=150). Other studies with a maximum age range of 20-29 years found comparable results, including one by Ojha N et al. (66%, N=57) on an unsupervised group [6] and another by Thaker RV et al. (54%, N=37) [7]. This age group, being more sexually active, is at a larger risk of unwanted pregnancies, thereby explaining the higher incidence of unsupervised MTP pill intake. This highlights the critical need for awareness programs tailored specifically to this age group. The high incidence of unsupervised MTP pill use among lower-income women suggests that there is a need to improve healthcare access, reproductive health education, and access to affordable and effective family planning services in marginalized areas. A review by Sanneving et al. [8] highlights the inequity of reproductive healthcare access among women, emphasizing that poor women are disproportionately affected. This disparity in healthcare distribution is visible whether the poor are urban or rural. In our study, husbands advocated for the use of MTP pills in 57.33% (N=150) of cases. This shows the significant influence husbands have on women's reproductive decisions. A study by Burrell et al. demonstrated the disparity between women and men in seeking sexual health knowledge (70.3% vs 52.1%, N=388) [9]. This highlights the importance of including men in reproductive health discussions and education.

Despite clear guidelines that these pills can only be prescribed by a person authorized under the MTP act and taken under medical supervision, the pills were obtained directly from pharmacies in 90% (N=150) of cases in our study. Similar results were reported by other studies such as Bajwa SK et al. (29.61%, N=260) [10] and Agarwal M et al. (76.66%, N=30) [11]. Alarmingly, 97.3% (N=150) of women were unaware of the risks associated with taking MTP pills. Additionally, the patients followed a variety of self-designed regimens. A high risk of life-threatening complications and increased failure rates are seen with inadequate or improper usage of MTP pills [12]. Given the low adherence to the required regimen (84%, N=150) among those who used MTP pills in our study, individuals who choose this method should be followed up on and monitored to ensure its effectiveness and safety. This can only be achieved by collaboration with dedicated and accessible healthcare services, coupled with sexual health awareness.

It is critical to recognize the limitations of our research. The cross-sectional design and a relatively small sample size with geographical limitations highlight the need for further large sample studies. In addition, using self-reported data may introduce recall bias. Despite these limitations, our research lays the groundwork for future efforts to reduce the number of unsafe abortions, promote safer reproductive health practices, and improve the well-being of women in our study population and similar settings around the world.

## Conclusions

In conclusion, this study has illuminated critical issues surrounding unsupervised MTP pill intake among women seeking healthcare at a tertiary care hospital in India. The findings highlight the necessity of interventions that focus not only on improving access to safe abortion services but also on addressing the underlying social and economic determinants influencing women's reproductive choices. There is a need for a multifaceted approach, which may include comprehensive reproductive health education, enhanced regulatory measures for MTP pills, and improved accessibility and affordability of healthcare services.
